# Health related quality of life in a nationally representative sample of haematological patients

**DOI:** 10.1111/j.1600-0609.2009.01250.x

**Published:** 2009-08

**Authors:** Anna T Johnsen, Dorte Tholstrup, Morten Aa Petersen, Lise Pedersen, Mogens Groenvold

**Affiliations:** 1 Department of Palliative Medicine, Bispebjerg HospitalCopenhagen NV; 2 Department of Haematology, RigshospitaletBlegdamsvej, Copenhagen OE, Denmark; 3 Institute of Public Health, University of CopenhagenOester Farimagsgade, Copenhagen K, Denmark

**Keywords:** quality of life, symptomatology, questionnaire, leukaemia, multiple myeloma, malignant lymphoma, haematologic diseases

## Abstract

**Objectives:**

Knowledge of health related quality of life of haematological patients is limited. This study aimed at investigating the prevalence and predictors of symptoms and problems in a representative sample of haematological patients in Denmark.

**Methods:**

A random sample of patients with leukaemia, multiple myeloma and advanced lymphoma (*n* = 732) received the European Organisation for Research and Treatment of Cancer quality-of-life questionnaire (EORTC QLQ-C30). Mean scores were calculated. In addition, scores were dichotomised using two thresholds: patients reporting at least ‘a little’ of each EORTC QLQ-C30 symptom/problem were classified as having a ‘symptom/problem’, and patients reporting at least ‘quite a bit’ were classified as having a ‘severe symptom/problem’. Multiple logistic regression was used to identify predictors.

**Results:**

In total, 470 (64%) patients participated. The most frequent symptoms/problems were fatigue (55%; severe 20%), reduced role function (49%; severe 23%), insomnia (46%; severe 15%), and pain (37%; severe 15%). Older patients and patients in active antineoplastic treatment had more symptoms and problems. There was only little evidence of social inequalities.

**Conclusion:**

This is probably the first nationally representative study of symptoms and problems in haematological patients. These patients have symptoms/problems that deserve attention. Health related quality of life is an important issue in haematological malignancies.

About 7% of all cases of cancer in Denmark are haematological malignancies (1). The diagnosis of a haematological malignancy and its treatment potentially lead to symptoms and problems that affect quality of life. An important task of the health care system is to assess, monitor, and prevent such symptoms and problems in order to help patients live as fully as possible with their disease (2, 3). This has only become more relevant as the survival has increased (4, 5), and the task is particularly important for diseases that may not be curable (6).

Despite a general acknowledgement of the importance of the patients’ health related quality of life (HRQOL) relatively few studies have investigated this issue in haematological patients (4, 6–9). In 2005 a review found only eight studies investigating HRQOL in patients with chronic lymphocytic leukaemia (CLL), which is the most common leukaemia in adults. None of the identified studies investigated patients who had never received treatment, measured quality of life in a random sample of CLL patients, or had quality of life as their primary objective (10). Potential reasons for the lack of studies of HRQOL in patients with CLL may be that many of these patients are expected to live a quite normal life (11), and that a large proportion of these patients are observed untreated or receive a treatment of moderate toxicity, and HRQOL outcomes may therefore be deemed less important (12). The picture of few studies is the same for acute leukaemia, but for these patients the reason may be that the diseases are very acute and therefore HRQOL studies are perhaps not considered feasible (8, 13). Similarly only few studies of HRQOL have been conducted for multiple myeloma (14).

Most studies of HRQOL in haematological patients have either focused on long-term survivors or have been part of clinical trials investigating different treatment regimens. To our knowledge, there exist no study assessing HRQOL in the entire group of haematological patients seen in hospitals. Such a study would provide clinically valuable information. It would specifically answer the two simple questions: to what extents are haematological patients burdened by symptoms and problems that deserve attention? And which groups of patients suffer from which symptoms and problems?

This study was part of a larger study assessing symptoms and problems in patients with advanced cancer defined as patients with TNM stages 3 or 4 (TNM: system for classifying cancers; T = local tumour growth, N = regional lymph nodes, M = metastasis) (the results concerning patients with solid tumours will be reported elsewhere). However, the TNM system is not fully applicable to haematological malignancies. Thus, the TNM system was employed for lymphoma but not for patients with leukaemia and multiple myeloma.

In the present study we therefore investigated HRQOL of adult patients with lymphoma in stage 3 or 4, and patients with multiple myeloma or leukaemia in any stage. The study was conducted as a nationally representative, cross-sectional study with the following aims: (i) to measure symptoms and problems; and (ii) to identify predictors of symptoms and problems.

## Method

### Patients

All clinical hospital departments (except for departments of psychiatry and paediatrics) from three out of 14 regions across Denmark (total population 5.5 millions) were invited to participate (*n* = 81). The three regions were selected to represent Denmark concerning geographic and demographic characteristics. In each participating department all cancer patients born from the 1st to the 22nd in the month were identified from the patient register. An exception was made for four departments where a smaller proportion of patients were selected (fewer birth days were included). This leads to a slight under-representation of patients from these departments, but since only few patients with haematological malignancies were identified from these departments this has no practical implications. Only patients who had been in contact with the hospital department within the past year, and lived in one of the three regions were retrieved from the registers.

Medical students reviewed the medical records of all patients in the retrievals. Patients were included if they had lymphoma stage 3 or 4 or leukaemia or multiple myeloma. Patients were excluded if they did not speak Danish, had a cognitive impairment or had a psychiatric co-morbidity.

Included patients received an information letter, a consent form, and a questionnaire by mail. A reminder was sent after 2 weeks. The study was approved by the local ethics committee (01-116/03 and 11-143/03) and took place from October 2004 to January 2006.

### Clinical data and assessments

The following clinical data were extracted from the medical records: gender, age, diagnosis, stage of disease for lymphomas [using the American Joint Committee on Cancer (AJCC) manual (15)], time of (first) diagnosis, contact type (latest contact with the hospital: out-patient or hospitalised), and treatment status (in active antineoplastic treatment or not).

Participants received the EORTC QLQ-C30 (16, 17) questionnaire assessing health-related quality of life. It consists of five multi-item function scales: physical, role, emotional, cognitive, and social function; a global health status/quality of life (QOL) scale; three multi-item symptom scales: fatigue, nausea and vomiting, and pain; and six single-item symptom scales: dyspnoea, insomnia, appetite loss, constipation, diarrhoea and financial difficulties. For the five function scales and global health status/QOL higher scores represent better functioning. Conversely, for the nine symptom scales higher scores represent more symptoms.

### Statistics

The analyses were performed using sas statistical software version 9 (SAS Institute Inc., Cary, NC, USA) (18). Participants and non-participants were compared using Wilcoxon (age, time since diagnosis), chi-square (region, type of departments, diagnosis) and Fisher’s exact tests (gender, treatment status, contact type).

**Figure 1:**
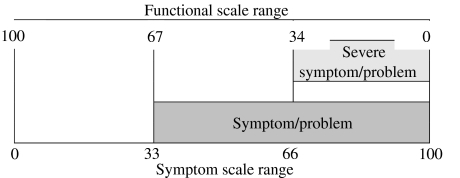
Illustration of definition of ‘symptom/problem’ and ‘severe symptom/problem’.

The responses to the EORTC QLQ-C30 were converted into 0–100 scales according to the scoring manual, and mean scores were calculated (17). For the EORTC QLQ-C30 there are no predefined thresholds for when a symptom or function score should be interpreted as a *case*. However, to ease clinical interpretation of the data, and in accordance with previous procedures (19), we dichotomised the scores using two thresholds: (i) we calculated frequencies of ‘symptoms/problems’ and defined that a patient had a ‘symptom/problem’ if the scale score corresponded to at least ‘a little’ (symptom scale ≥ 33, function scale ≤ 67); and (ii) we calculated frequencies of ‘severe symptoms/problems’ and defined that a patient had a ‘severe symptom/problem’ if the score corresponded to at least ‘quite a bit’ (symptom scale ≥ 66, function scale ≤ 34) (see Fig. 1). This was done for all scales except global health status/QOL. Finally, we calculated the number of ‘symptoms/problems’ and ‘severe symptoms/problems’ for each person. These numbers include both symptom scales and function scales, but exclude the global health status/QOL scale, and thus range from 0–14.

We used ordinal logistic regression to identify the predictors of symptoms and loss of function. We used a stepwise procedure with inclusion/exclusion criteria of *P* = 0.01. The following clinical and sociodemographic variables were tested: gender, age, diagnosis, time since diagnosis, contact type, treatment status, type of department, region, formal education, children and marital status.

## Results

### Patients

**Figure 2:**
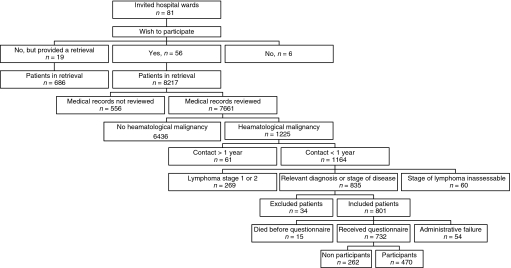
Inclusion of patients.

Figure 2 shows the inclusion process. In total, 56 (69%) of the invited hospital departments participated (27 departments of surgery, 23 departments of medicine, four departments of oncology and two departments of haematology), and from their patient registers we were provided with lists of 8217 patients. From 19 of the 25 non-participating departments we were provided with retrievals containing 686 additional patients. Hence, we could have reviewed 8% more medical records if they had participated too. None of the non-participating departments were departments of haematology, oncology, or internal medicine. We reviewed the medical records of 7661 of the 8217 patients. The remaining 556 (7%) medical records were not reviewed because they were impossible to get a hold on after several attempts (e.g. the medical record was misplaced or stored in an achieve another place).

Of the 7661 medical records reviewed, 1225 patients were identified as having a haematological malignancy. Of these, we excluded 61 because they had not had contact to the department within the previous year; 269 because they had lymphoma stage 1 or 2; 60 because stage of lymphoma was inaccessible; and 34 because they had a cognitive impairment, had a psychiatric illness, or did not speak Danish. Of the 801 patients included, 54 did not receive a questionnaire because of administrative failure, and 15 died before receiving a questionnaire. Thus, questionnaires were sent to 732 patients representing 19 hospital departments. In total, 470 (64%) patients returned a completed questionnaire.

Characteristics of participants and non-participants can be seen in Table 1. Compared to non-participants, participants were younger (mean age 63 years vs. 66 years), and were less likely to have a diagnosis of CLL and non-Hodgkin’s lymphoma.

**Table 1:** Characteristics of participants and non-participants

		Participants		Non-participants		
Characteristics		*N*	%	*N*	%	*P*-value
No. of patients		470	64	262	36	
Age, mean		470	63	262	66	0.02
Gender	Male	248	53	133	51	0.70
	Female	222	47	129	49	
Primary tumour site	AML	34	7	9	4	0.02
	CLL	132	28	92	35	
	CML	34	7	8	3	
	Hodgkin lymphoma	33	7	11	4	
	Non-Hodgkin lymphoma	164	35	102	39	
	Multiple myeloma	54	11	32	12	
	Other^1^	19	4	8	3	
Time since diagnosis	0–6 months	57	13	23	9	0.45
	6–12 months	35	8	23	9	
	1–2 years	64	14	49	20	
	2–5 years	124	28	56	22	
	5–10 years	111	25	61	24	
	>10 years	59	13	38	15	
Ongoing treatment	Yes	99	22	59	23	0.71
	No	358	78	199	77	
Contact type	Hospitalised	24	5	19	7	0.25
	Out-patients	439	95	242	93	
Department	Haematological	405	86	222	84	0.70
	Medical	52	11	34	13	
	Other^2^	13	3	6	2	
Region	Copenhagen	206	44	121	46	0.19
	Ringkoebing	32	7	26	10	
	Funen	232	49	115	44	
Formal education^3^	None	66	17	–		–
	Semi-skilled worker/short education (<1 year)	52	13	–		
	Skilled worker	39	10	–		
	Short theoretical (1–3 years)	57	15	–		
	Long theoretical (>3 years)	133	34	–		
	Academic	46	12	–		
Civil status^3^	Married/cohabiting	291	63	–		–
	Divorced/separated	54	12	–		
	Unmarried	46	10	–		
	Widow/widower	68	15	–		
Children^3^	Yes	386	84	–		–
	No	76	16	–		

CLL, chronic lymphocytic leukaemia; CML, chronic myeloid leukaemia; AML, acute myeloid leukaemia.

1 Patients with ALL, myelofibrosis or unclassified leukaemia.

2 Patients from departments of surgery and oncology.

3 These variables were assessed in participants only.

### Symptoms and problems

Table 2 shows the mean scores for the symptom and function scales, and the percentages of patients having a ‘symptom’ or a ‘severe symptom’ for the total sample and for each diagnosis. The symptom with the highest mean score (indicating most symptoms) was fatigue, followed by pain and insomnia. The function scale with the lowest mean score (indicating the most severe impairment) was role function, followed by physical function. Using the cut-off values, 55% of the patients had fatigue (20% severely), 49% reduced role function (23% severely), 46% insomnia (15% severely), and 37% pain (15% severely). The mean number of ‘symptoms/problems’ was 4.3, and the mean number of ‘severe symptoms/problems’ was 1.5. In total, 82% of the patients had at least one ‘symptom/problem’, and 45% had at least one ‘severe symptom/problem’.

**Table 2:** Mean scores and prevalences for the total sample and by diagnosis

Diagnosis		Physical function	Role function	Emotional function	Social function	Cognitive function	Quality of life	Pain	Fatigue	Nausea/ emesis	Dyspnoea	Appetite loss	Insomnia	Diarrhoea	Constipation	Financial difficulties	Mean no. of ‘symptoms’	Mean no. of ‘severe symptoms’
Total *N* = 470	Mean score	77	69	82	82	81	67	23	35	5	18	13	22	10	9	10	4.3	1.5
	% symptoms	34%	49%	24%	30%	31%		37%	55%	8%	36%	25%	46%	21%	20%	19%		
	% severe symptoms	9%	23%	5%	10%	9%		15%	20%	2%	15%	11%	15%	6%	6%	7%		
AML *N* = 34	Mean score	86	75	79	85	79	74	10	34	5	12	12	19	7	2	10	3.7	0.9
	% symptoms	18%	38%	30%	30%	33%		24%	47%	6%	24%	24%	56%	15%	6%	21%		
	% severe symptoms	3%	18%	3%	3%	12%		3%	18%	0%	6%	12%	0%	6%	0%	6%		
CLL *N* = 132	Mean score	76	71	84	85	82	67	22	35	5	21	12	25	11	11	6	4.0	1.4
	% symptoms	36%	46%	21%	21%	27%		37%	55%	8%	40%	26%	48%	22%	23%	14%		
	% severe symptoms	8%	22%	4%	10%	8%		14%	22%	2%	19%	8%	18%	8%	7%	4%		
CML *N* = 34	Mean score	81	71	85	83	82	71	25	33	10	22	14	20	11	5	8	4.2	1.5
	% symptoms	26%	45%	25%	31%	25%		32%	53%	21%	44%	29%	35%	31%	13%	16%		
	% severe symptoms	6%	21%	6%	3%	9%		24%	18%	0%	21%	12%	18%	3%	3%	6%		
Hodgkin lymphoma *N* = 33	Mean score	80	73	82	78	75	68	14	36	4	20	11	22	5	5	10	3.9	1.7
	% symptoms	27%	45%	22%	38%	41%		21%	48%	6%	39%	18%	42%	13%	13%	13%		
	% severe symptoms	12%	21%	9%	13%	16%		9%	24%	0%	15%	9%	18%	3%	3%	9%		
Non-Hodgkin lymphoma *N* = 164	Mean score	78	73	83	84	82	68	23	33	5	16	11	22	8	9	11	4.1	1.3
	% symptoms	34%	44%	24%	28%	30%		38%	55%	8%	33%	21%	46%	20%	21%	19%		
	% severe symptoms	7%	19%	4%	9%	7%		15%	16%	2%	13%	9%	16%	4%	5%	9%		
Multiple myeloma *N* = 54	Mean score	66	49	82	72	77	61	34	45	6	17	18	21	12	16	15	5.6	2.3
	% symptoms	54%	80%	22%	46%	33%		50%	67%	7%	37%	30%	44%	24%	33%	31%		
	% severe symptoms	15%	44%	7%	19%	15%		24%	26%	4%	9%	20%	13%	9%	11%	9%		
Other *N* = 19	Mean score	77	66	70	76	80	61	22	40	3	22	22	28	14	9	11	4.9	2.2
	% symptoms	28%	50%	47%	42%	47%		42%	56%	0%	33%	33%	56%	16%	16%	21%		
	% severe symptoms	17%	22%	16%	21%	0%		11%	28%	0%	28%	22%	17%	16%	11%	11%		

CLL, chronic lymphocytic leukaemia; CML, chronic myeloid leukaemia; AML, acute myeloid leukaemia.

% symptoms, proportion of patients scoring at most 67 for function scales or at least 33 for symptom scales.

% severe symptoms, proportion of patients scoring at most 34 for function scales or at least 66 for symptom scales.

No. of ‘symptoms’, mean number of symptoms using the same cut-point as in ‘% symptoms’ (quality of life excluded).

No. of ‘severe symptoms’, mean number of symptoms using the same cut-point as in ‘% severe symptoms’ (quality of life excluded).

There were significant differences in the levels of physical function, role function, social function, pain, and constipation across the diagnoses (*P*-values not shown in Table 2). Generally, patients with multiple myeloma had most symptoms and problems. The mean number of ‘symptoms/problems’ ranged from 3.7 (AML) to 5.6 (multiple myeloma); ‘severe symptoms/problems’ ranged from 0.9 (AML) to 2.3 (multiple myeloma).

### Predictors

Results of the multivariate logistic regressions can be seen in Table 3. Older patients were more reduced in physical and role function, had lower quality of life, and more constipation, appetite loss and pain than younger patients, but less financial difficulties. Women had lower physical function, and more insomnia than men. Recently hospitalised patients had more nausea and appetite loss than out-patients. Patients in active treatment had more reduced physical function, lower quality of life, and more appetite loss and fatigue than patients not in active treatment. Patients from departments of medicine had lower quality of life and more fatigue, than patients from haematological departments. Diagnosis was only a significant predictor of one scale in the multivariate analysis: patients with multiple myeloma had more reduced role function. Finally, patients with shorter education (as opposed to longer education) reported more reduced cognitive function (see Table 3).

**Table 3:** Predictors of symptoms and problems of the EORTC QLQ-C30 using ordinal logistic regression

Scale	Predicting variable(s)^1^	OR^2^	(95% CL)	*P*
Physical function	Age (per 10 year)	1.53	(1.36–1.74)	<.001
	Sex (male vs. female)	0.58	(0.42–0.80)	0.001
	Active treatment (yes vs. no)	1.87	(1.26–2.79)	0.002
Role function	Age (per 10 year)	1.32	(1.16–1.51)	<.001
	Diagnosis:			0.002
	CLL	1.01	(0.66–1.55)	
	CML	1.24	(0.62–2.47)	
	AML	1.14	(0.56–2.31)	
	Hodgkin lymphoma	1.36	(0.67–2.78)	
	Non-Hodgkin lymphoma	1.00	–	
	Multiple myeloma	3.25	(1.86–5.67)	
	Other	2.03	(0.83–4.97)	
Emotional function	No significant predictors			
Social function	No significant predictors			
Cognitive function	Education:			
	None	1.79	(0.94–3.42)	0.005
	Semi-skilled worker (<1 yr)	1.23	(0.62–2.45)	
	Skilled worker	1.02	(0.49–2.17)	
	Short theoretical (1–3 years)	1.00	–	
	Long theoretical (>3 years)	0.71	(0.40–1.28)	
	Academic	0.51	(0.24–1.08)	
	Age (per 10 year)	1.18	(1.05–1.32)	0.006
Quality of life	Active treatment (yes vs. no)	1.96	(1.31–2.92)	0.001
	Department:			
	Haematology	1.00	–	0.007
	Medicine	2.13	(1.23–3.69)	
	Other	2.43	(0.92–6.34)	
Fatigue	Active treatment (yes vs. no)	1.85	(1.25–2.74)	0.002
	Department:			
	Haematology	1.00	–	0.006
	Medicine	1.82	(1.06–3.12)	
	Other	3.43	(1.30–9.05)	
Pain	Age (per 10 year)	1.28	(1.13–1.45)	<.001
Nausea/vomiting	Contact type (hospitalised vs. out-patient)	2.98	(1.30–6.83)	0.010
Dyspnoea	No significant predictors			
Appetite loss	Age (per 10 year)	1.28	(1.08–1.51)	0.004
	Active treatment (yes vs. no)	2.03	(1.25–3.29)	0.004
	Contact type (hospitalised vs. out-patient)	3.14	(1.33–7.41)	0.009
Insomnia	Sex (male vs. female)	0.46	(0.33–0.66)	<.001
Diarrhoea	No significant predictors			
Constipation	Age (per 10 year)	1.47	(1.22–1.78)	<.001
Financial difficulties	Age (per 10 year)	0.76	(0.65–0.89)	<.001

CL, confidence limits; CLL, chronic lymphocytic leukaemia; CML, chronic myeloid leukaemia; AML, acute myeloid leukaemia.

1 Variables in the final model of the logistic regression.

2 An odds ratio above 1 reflects more functional limitations or more symptoms.

## Discussion

This nationally representative, cross-sectional study assessed a random sample of patients with multiple myeloma, leukaemia or lymphoma (stage 3 or 4) seen at Danish hospitals, mainly departments of haematology at university hospitals, within the previous year. The study showed that most of the symptoms and problems assessed were frequent. Using the cut-off values the most prevalent symptoms and problems were fatigue (55%; severe 20%), reduced role function (49%; severe 23%), insomnia (46%; severe 15%), pain (37%; severe 15%), and dyspnoea (36%; severe 15%). This indicates that efforts to improve the management should be given high priority.

The figures above do not take into consideration the potential co-morbidity of the patients. As such, the figures show the symptoms and problems experienced by this population regardless of their aetiology, and thus, of course, not everything can be attributed to a haematological disease.

No previous studies have included a patient sample similar to ours, and therefore comparisons are difficult. A study of CLL patients who were not receiving current anti cancer treatment (12) showed similar levels of symptoms and problems as the CLL patients in our study, al though the women of that study generally had higher levels of symptoms and problems whereas the males generally had lower levels.

In a study of AML patients (13) the reported levels of symptoms and problems at the end of in-patient treatment were similar to those reported by the AML patients in our study. However, our patients had less appetite loss, nausea and dyspnoea, and better social function. This may reflect that some of the patients included in our study had been without treatment for a long time.

A Norwegian study of Hodgkin’s lymphomas survivors (20) found levels of symptoms and problems that were generally lower than found for the Hodgkin group in our study. This probably reflects that our study only included patients with advanced lymphomas. In addition, our study included patients in all disease phases and thus, some of the patients’ experienced treatment-related symptoms. However, the level of dyspnoea and constipation was higher in the Norwegian study.

A Nordic study of patients with multiple myeloma (14) generally showed higher levels of symptoms and problems before patients began treatment than showed for the myeloma patients in the present study (14). The level of symptoms and problems after 6 months of treatment reported in the study by Wisloffs *et al.*(14) were similar to our findings.

It is difficult to interpret our results by comparing them with the few, relevant other studies conducted, since there are substantial differences in the sampling of patients. The most important way to interpret our results, from a clinical perspective, is probably done by a careful examination of the prevalences of symptoms and problems in the various subgroups.

### Predictors of symptoms and problems

Age was a strong predictor of several symptom and function scales with older patients having more symptoms and problems. This is consistent with findings from general populations, where older age is associated with more symptoms and problems (21–23), and older age has also been associated with lower quality of life in two studies of CLL patients (6, 12). In contrast, studies of advanced cancer patients have generally found that younger patients are more burdened by symptoms and problems (24–28), and younger patients reported more depression and worse emotional and social function in a study of CLL patients (11). Thus, results of the present study draw attention to the symptom-burden of the older patients, but the social and emotional strain of disease of younger patients should not be forgotten.

Patients in active treatment had more symptoms and problems. This is consistent with previous findings in haematology (29), and in cancer in general (25, 30).

Recently hospitalised patients had more nausea and appetite loss than out-patients, which is an important finding. It indicates that identification and treatment of these symptoms for especially hospitalised patients is needed.

Patients with multiple myeloma generally had the highest level of symptoms and problems. This may reflect that multiple myeloma is characterised by distress due to bone pain, pathological bone fractures and recurrent infections (14). However, in the multivariate analysis, patients with multiple myeloma only were significantly more reduced in role function. A possible reason may be, that patients with multiple myeloma generally were older than patients with all other diagnoses (except CLL), and older age showed to be a strong predictor. Patients diagnosed with AML showed the lowest level of symptoms and problems. This is consistent with the findings from a study that compared patients with acute leukaemia to patients with advanced Hodgkin’s lymphoma 5 years after treatment, and found that Hodgkin’s patients had significantly greater psychological distress and greater fatigue (31).

Women reported more reduced physical function, and had more insomnia than men. This is consistent with findings from general populations (21–23), and has also been found in CLL patients (12, 29), and in adult leukaemia survivors (32). However, a study of Hodgkin’s lymphomas survivors found that women had less fatigue and better quality of life (20).

Previous studies have shown that highly educated patients have fewer symptoms and problems (24, 25, 30, 33), and in a study of adult leukaemia survivors patients with less education reported worse overall adjustment (32). Surprisingly, education was related to cognitive function only in this study. Hence, the study only partially supports the notion that there are social inequalities in the prevalence of symptoms and problems. It has previously been found that unmarried patients with advanced cancer have more symptoms and problems (25, 26), but this was neither supported in the present study nor in a study of adult leukaemia survivors (32).

### Strengths and limitations

A major strength of this study is that we included an almost representative, national sample of patients with lymphoma (stage 3 or 4), leukaemia and multiple myeloma who have been in contact with a hospital department within the previous year. The participation of hospital departments was relatively high: all haematological departments in the selected regions participated, and we reviewed the majority of the medical records from the patients identified. However, there is an under-representation of patients coming from the rural region ‘Ringkoebing’ because some patients from this region are referred to a specialised department of haematology in a region not included in the study.

Given the representativeness of the patients included, the study gives a picture of the burden of symptoms and problems in haematological patients in contact with a hospital department. The important message is that these patients do have symptoms and problems that do deserve attention and resources in the health care system.

There are three primary weaknesses of the study. First, not all patients participated. A response rate of 64% is not optimal. However, it is within the range of what is often found in cross-sectional surveys with mailed self-assessment questionnaires (11, 34–36) and it is impossible to avoid some non-participation in such studies. If patients had been approached in person and/or we had used structured interviews we might have achieved a better response rate, but this was not feasible with our design. The oldest patients participated least in the study and together with the fact that the most burdened patients are the ones least likely to participate, this means that the study probably underestimates symptoms and problems. However, this does not jeopardise the primary conclusion of the paper: that there is a substantial prevalence of symptoms and problems in this group of patients.

Second, the fact that we included patients irrespective of diagnoses, treatment, etc. made it more difficult to subdivide patients in clinically well characterised subgroups than if a highly selected subgroup of patients was included. For example, we found it impossible to devise classifications of treatments that were applicable across the diverse spectrum of diagnoses and treatment phases. Knowledge about the effect of specific treatments on quality of life must be obtained from studies designed for this.

Third, due to space limitations in the questionnaire (we were afraid the response burden was already significant) we did not collect data on co-morbidity, which would have been beneficial, and it is a limitation of the study that we are not able to control for this potential confounder. On the other hand, for the primary purpose of this study, co-morbidity did not seem crucial. Patients need attention to and care for their experienced symptoms and problems regardless of aetiology.

## Conclusion

This is probably the first nationally representative study of symptoms, problems and quality of life in haematological patients. These patients have symptoms and problems that deserve attention, and older patients and patients undergoing treatment are especially burdened. The study found little evidence of social inequalities in HRQOL. The study indicates that haematological malignancies affect HRQOL broadly and points to the challenge of alleviating this impact on HRQOL.

## References

[1] Clemmensen I, Nedergaard KH, Storm HH (2006). [Cancer in Denmark] [in Danish].

[2] Fagerstrom L (1998). The patient’s perceived caring needs as a message of suffering. J Adv Nurs.

[3] Foot G, Sanson-Fisher R (1995). Measuring the unmet needs of people living with cancer. Can Forum.

[4] Zittoun R (1993). Quality of life in hematological malignancies with brief reference to bone marrow transplantation. Leuk Res.

[5] Lim JW, Zebrack B (2006). Social networks and quality of life for long-term survivors of leukemia and lymphoma. Support Care Cancer.

[6] Molica S (2005). Quality of life in chronic lymphocytic leukemia: a neglected issue. Leuk Lymphoma.

[7] Montgomery C, Pocock M, Titley K, Lloyd K (2002). Individual quality of life in patients with leukaemia and lymphoma. Psychooncology.

[8] Efficace F, Novik A, Vignetti M, Mandelli F, Cleeland CS (2007). Health-related quality of life and symptom assessment in clinical research of patients with hematologic malignancies: where are we now and where do we go from here?. Haematologica.

[9] Efficace F, Kemmler G, Vignetti M, Mandelli F, Molica S, Holzner B (2008). Health-related quality of life assessment and reported outcomes in leukaemia randomised controlled trials – a systematic review to evaluate the added value in supporting clinical decision making. Eur J Cancer.

[10] Stephens JM, Gramegna P, Laskin B, Botteman MF, Pashos CL (2005). Chronic lymphocytic leukemia: economic burden and quality of life: literature review. Am J Ther.

[11] Levin TT, Li Y, Riskind J, Rai K (2007). Depression, anxiety and quality of life in a chronic lymphocytic leukemia cohort. Gen Hosp Psychiatry.

[12] Holzner B, Kemmler G, Kopp M, Nguyen-Van-Tam D, Sperner-Unterweger B, Greil R (2004). Quality of life of patients with chronic lymphocytic leukemia: results of a longitudinal investigation over 1 yr. Eur J Haematol.

[13] Schumacher A, Kessler T, Buchner T, Wewers D, van de LJ (1998). Quality of life in adult patients with acute myeloid leukemia receiving intensive and prolonged chemotherapy – a longitudinal study. Leukemia.

[14] Wisloff F, Eika S, Hippe E, Hjorth M, Holmberg E, Kaasa S, Palva I, Westin J (1996). Measurement of health-related quality of life in multiple myeloma. Nordic Myeloma Study Group. Br J Haematol.

[15] American joint committee on cancer (2002). AJCC – Cancer Staging Manual.

[16] Aaronson NK, Ahmedzai S, Bergman B (1993). The European-organization-for-research-and-treatment-of-cancer qlq-c30 - a quality-of-life instrument for use in international clinical-trials in oncology. J Natl Cancer Inst.

[17] Fayers P, Aaronson NK, Bjordal K, Groenvold M, Curran D, Bottomley A (2001). The EORTC QLQ-C30 Scoring Manual.

[18] SAS Institute Inc (2004). SAS/STAT 9.1 User’s Guide.

[19] Stromgren AS, Goldschmidt D, Groenvold M, Petersen MA, Jensen PT, Pedersen L, Hoermann L, Helleberg C, Sjogren P (2002). Self-assessment in cancer patients referred to palliative care: a study of feasibility and symptom epidemiology. Cancer.

[20] Norum J, Wist EA (1996). Quality of life in survivors of Hodgkin’s disease. Qual Life Res.

[21] Hjermstad MJ, Fayers PM, Bjordal K, Kaasa S (1998). Health-related quality of life in the general Norwegian population assessed by the European Organization for Research and Treatment of Cancer Core Quality-of-Life Questionnaire: the QLQ=C30 (+3). J Clin Oncol.

[22] Schwarz R, Hinz A (2001). Reference data for the quality of life questionnaire EORTC QLQ-C30 in the general German population. Eur J Cancer.

[23] Yun YH, Kim SH, Lee KM, Park SM, Kim YM (2007). Age, sex, and comorbidities were considered in comparing reference data for health-related quality of life in the general and cancer populations. J Clin Epidemiol.

[24] Gil KM, Gibbons HE, Jenison EL, Hopkins MP, von Gruenigen VE (2007). Baseline characteristics influencing quality of life in women undergoing gynecologic oncology surgery. Health Qual Life Outcomes.

[25] Parker PA, Baile WF, de Moor C, Cohen L (2003). Psychosocial and demographic predictors of quality of life in a large sample of cancer patients. Psychooncology.

[26] King MT, Kenny P, Shiell A, Hall J, Boyages J (2000). Quality of life three months and one year after first treatment for early stage breast cancer: influence of treatment and patient characteristics. Qual Life Res.

[27] Lundh HC, Seiger A, Furst CJ (2006). Quality of life in terminal care – with special reference to age, gender and marital status. Support Care Cancer.

[28] Walsh D, Donnelly S, Rybicki L (2000). The symptoms of advanced cancer: relationship to age, gender, and performance status in 1,000 patients. Support Care Cancer.

[29] Shanafelt TD, Bowen D, Venkat C, Slager SL, Zent CS, Kay NE, Reinalda M, Sloan JA, Call TG (2007). Quality of life in chronic lymphocytic leukemia: an international survey of 1482 patients. Br J Haematol.

[30] Jordhoy MS, Fayers P, Loge JH, Saltnes T, Ahlner-Elmqvist M, Kaasa S (2001). Quality of life in advanced cancer patients: the impact of sociodemographic and medical characteristics. Br J Cancer.

[31] Kornblith AB, Herndon JE, Zuckerman E (1998). Comparison of psychosocial adaptation of advanced stage Hodgkin’s disease and acute leukemia survivors. Cancer and Leukemia Group B. Ann Oncol.

[32] Greenberg DB, Kornblith AB, Herndon JE, Zuckerman E, Schiffer CA, Weiss RB, Mayer RJ, Wolchok SM, Holland JC (1997). Quality of life for adult leukemia survivors treated on clinical trials of Cancer and Leukemia Group B during the period 1971–1988: predictors for later psychologic distress. Cancer.

[33] van den Beuken-van Everdingen MH, de Rijke JM, Kessels AG, Schouten HC, van Kleef M, Patijn J (2007). High prevalence of pain in patients with cancer in a large population-based study in The Netherlands. Pain.

[34] Andrykowski MA, Bishop MM, Hahn EA, Cella DF, Beaumont JL, Brady MJ, Horowitz MM, Sobocinski KA, Rizzo JD, Wingard JR (2005). Long-term health-related quality of life, growth, and spiritual well-being after hematopoietic stem-cell transplantation. J Clin Oncol.

[35] Barg FK, Cronholm PF, Straton JB, Keddem S, Knott K, Grater J, Houts P, Palmer SC (2007). Unmet psychosocial needs of Pennsylvanians with cancer: 1986–2005. Cancer.

[36] Anderson H, Ward C, Eardley A, Gomm SA, Connolly M, Coppinger T, Corgie D, Williams JL, Makin WP (2001). The concerns of patients under palliative care and a heart failure clinic are not being met. Palliat Med.

